# Boronic Acid Adsorption on Hydrated Rutile TiO_2_(110): A DFT + U Study

**DOI:** 10.1021/acsomega.5c07528

**Published:** 2025-11-13

**Authors:** Leah Isseroff Bendavid, Julie Geller

**Affiliations:** Department of Chemistry, 6711 Vassar College, 124 Raymond Ave, Box 175, Poughkeepsie, New York 12604, United States

## Abstract

Boronic acids have emerged as promising anchoring groups for dye-sensitized solar cells (DSSCs). While previous computational studies have examined their adsorption on clean, ideal TiO_2_ surfaces, real-world conditions often involve hydrated surfaces. In this work, we investigate the adsorption behavior of boric acid and five functionalized boronic acids on the hydrated rutile TiO_2_(110) surface, using density functional theory with the DFT + *U* Hubbard correction and D3 dispersion corrections. To represent the hydrated surface, five distinct models were constructed, each featuring a different geometry of low-coverage dissociative water adsorption. For each hydrated surface, we examined a variety of molecular and dissociative monodentate and bidentate adsorption configurations. Boric acid was found to preferentially adsorb in a bidentate, doubly dissociative configuration on the hydrated surface, consistent with its optimal binding mode on the clean surface. Hydration weakens the adsorption of boronic acids compared to the clean surface, but preserves the same trends in relative binding strength across functional groups—functionalization still enhances binding stability, with fluorophenylboronic acids showing the strongest adsorption. Bader charge analysis reveals that hydration decreases the positive charges on the Ti surface atoms, reducing their Lewis acidity, weakening adsorption, and diminishing the sensitivity of adsorption strength to substitution. This study provides a more realistic benchmark for boronic acid adsorption under ambient conditions and informs the future design of anchoring groups for TiO_2_-based applications.

## Graphical abstract

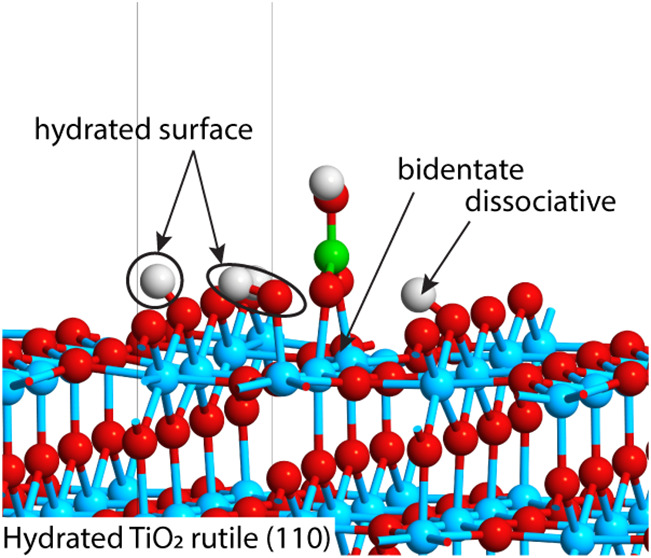

## Introduction

TiO_2_ is a wide-bandgap semiconductor with broad applications in photovoltaics, photocatalysis, and surface engineering. In dye-sensitized solar cells (DSSCs),^1^ nanostructured TiO_2_ serves as the semiconducting material that collects and transports photoexcited electrons, generating the photovoltage. The TiO_2_ surface is typically coated with a monolayer of dye molecules that absorb sunlight and inject photoexcited electrons into the TiO_2_ conduction band. For effective electron transfer and long-term operational stability, these dye molecules are functionalized with anchoring groups that chemisorb onto the TiO_2_ surface. The identity of the anchoring group plays a critical role in tuning the interfacial electronic structure, adsorption strength, and charge-transfer dynamics.

Carboxylic acids and their derivatives are the most widely used anchoring groups due to their well-established stability.^1,2^ Phosphonic acids have also been studied as more robust alternatives.^3–5^ More recently, boronic acids—including boric acid and substituted derivatives—have emerged as promising candidates for TiO_2_ surface functionalization.^6–9^

In a prior computational study,^10^ we examined the adsorption of boronic acids on the clean TiO_2_ rutile (110) surface to establish a reference framework for adsorption behavior. Beginning with the ideal surface was helpful for isolating the intrinsic binding preferences of each molecule—such as monodentate versus bidentate coordination and molecular versus dissociative adsorption—without interference from surface hydroxyl species, as well as to more clearly assess the impact of boronic acid functionalization. This approach also facilitated direct comparison with existing literature, which predominantly models adsorption on clean TiO_2_.^11^ It also established a benchmark that enables us to assess how surface modification changes adsorption strength and geometry.

However, studying boronic acid adsorption on the *hydrated* TiO_2_ rutile (110) surface is important for realistic insights into its potential as an anchoring group in DSSCs. In practical DSSC environments, the TiO_2_ surface is typically in contact with an electrolyte solution consisting of organic solvents, but may also be exposed to a humid environment during its synthesis or operate under humid conditions. Consequently, it may be partially hydrated at a low coverage, with water molecules or hydroxyl groups adsorbed on the surface. This can alter surface reactivity and local coordination geometry, affecting how boronic acids will bind with the surface. The present study therefore extends this analysis to the hydrated TiO_2_(110) surface, incorporating low-coverage dissociative water adsorption to better reflect surface conditions under ambient environments. We evaluate how hydration influences boronic acid binding stability and geometry, as well as how hydration changes the impact that functionalization has on strengthening binding.

In this work, we follow the same approaches established as best practice in our previous study.^10^ We investigate the same set of adsorbates—boric acid and boronic acids substituted with methyl, phenyl, and fluorophenyl groups. We employ periodic density functional theory (DFT) calculations using the DFT+U method^12^ to capture the localized d-electron behavior of Ti (which standard DFT fails to capture accurately) and with DFT-D3 dispersion corrections^13,14^ to model the noncovalent interactions important for surface adsorption. Hydrated TiO_2_ surfaces were modeled at low water coverage by constructing several distinct configurations containing dissociatively adsorbed water near the boronic acid anchoring site, to capture the most pronounced effects of surface hydration on adsorption behavior. A range of adsorption structures—including molecular and dissociative, monodentate and bidentate configurations—were sampled and optimized for boric acid, and a subset of the optimal configurations were then considered for the rest of the boronic acids.

By modeling boronic acid adsorption on hydrated surfaces, this work provides a more realistic and detailed understanding of boronic acid adsorption on TiO_2_ under operational conditions. The insights gained are expected to inform the selection and design of anchoring groups for DSSCs and other TiO_2_-based applications.

## Methods

### Structural Models

The rutile TiO_2_(110) surface was represented by a five-layer slab model, consistent with prior studies demonstrating convergence of surface properties at this thickness.^15^ Experimental lattice constants were used to construct the slab, with a surface cell replication of 4 × 2 (11.836 Å × 12.991 Å). This larger size of the surface has been shown to more accurately model adsorption of boronic acids.^10^ A vacuum gap of 20 Å was introduced along the surface normal to eliminate interactions between periodic slabs. During all structural optimizations involving a slab, lattice parameters were held fixed to the experimental values and atoms in the bottom layer were fixed to their bulk positions, while all other atoms were fully relaxed, to better converge the surface properties.

The rutile TiO_2_(110) surface (Figure 1) is composed of alternating rows of four distinct atomic species: 5-fold-coordinated titanium (Ti_5c_), 6-fold-coordinated titanium (Ti_6c_), in-plane 3-fold-coordinated oxygen (O_ip_), and bridging 2-fold-coordinated oxygen (O_b_). Among these, the undercoordinated Ti_5c_ and O_b_ atoms exhibit the highest reactivity. The Ti_5c_ sites preferentially coordinate with the oxygen atoms in adsorbates, while the O_b_ atoms can participate in hydrogen bonding interactions.

**Figure 1:**
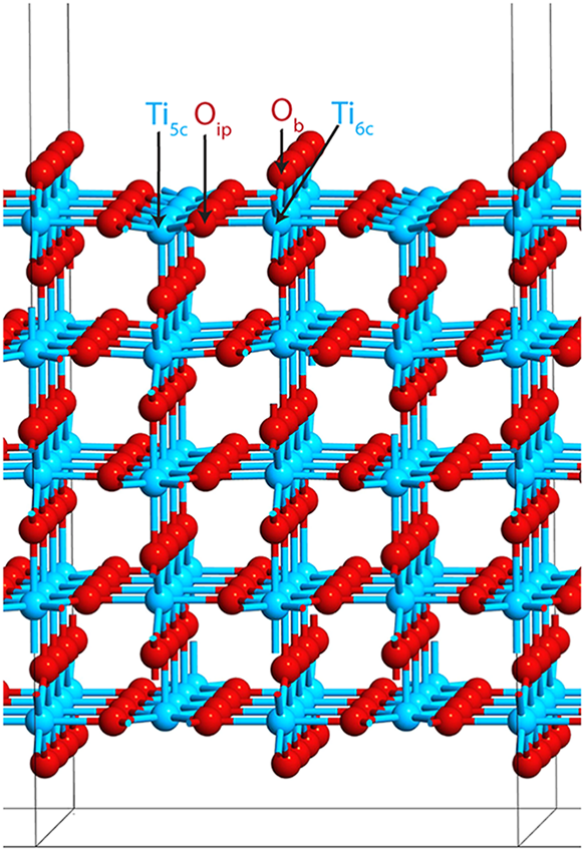
Rutile TiO_2_ (110) surface slab model, with the four types of unique surface atoms indicated.

In the DSSC environment, only a limited amount of water is present, either as residual trace water in the organic electrolyte or as hydroxyl species bound to the TiO_2_ surface from synthesis and handling. It is therefore appropriate to model the surface under conditions of low hydration. A low-coverage model avoids dense water overlayers that could mask the intrinsic binding sites of TiO_2_ and allows us to better isolate adsorbate–surface interactions. In this study, we represent hydration by adding a single water molecule per surface cell, corresponding to a coverage of 1/8 monolayer, which provides a computationally tractable and chemically relevant model of partial surface hydroxylation.

Water is known to adsorb dissociatively on the rutile TiO_2_(110) surface at low coverages.^16^ To model a partially hydrated surface, five distinct configurations (Figure 2) were constructed using the optimized large slab. In each configuration, the hydroxyl (OH^-^) and proton (H^+^) species resulting from water dissociation were positioned on a Ti_5c_ and an O_b_, respectively, near the expected adsorption sites of the boronic acids to capture local interactions. These five arrangements introduce structural diversity and allow assessment of how different hydration environments affect adsorption behavior. Configurations A1, A2, and A3 share the same hydroxyl adsorption site but differ in the location of the dissociated proton. Similarly, configurations B1 and B2 have a common hydroxyl adsorption site, with variations in proton placement. The binding site of the adsorbed boronic acid, common to all five surfaces, is circled in Figure 2. Optimization of the hydrated surfaces was found to be highly sensitive to the initial placement of protons. To promote stable convergence, hydrogen atoms were initially positioned directly above undercoordinated surface oxygen atoms or the oxygen atoms of hydroxyl groups.

**Figure 2:**
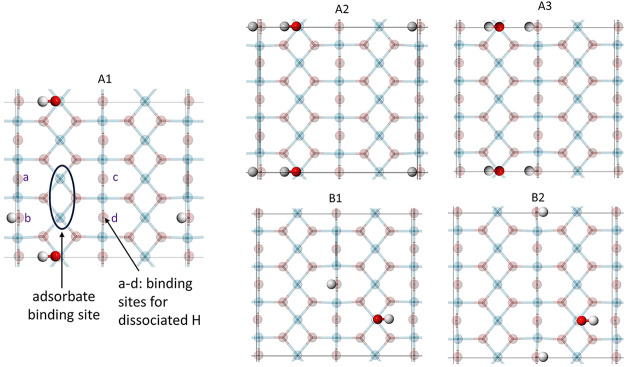
Top view of the five configurations used to model the hydrated rutile TiO_2_ (110) surface.

Boronic acids can adsorb onto TiO_2_ surfaces in either monodentate or bidentate configurations, in which one or two oxygen atoms from the molecule coordinate to Ti atoms on the surface (Figure 3). In the monodentate mode, additional stabilization may arise from hydrogen bonding between a hydrogen atom on the molecule and a nearby surface oxygen atom. In the bidentate configuration, the two oxygen atoms are expected to bridge between two adjacent Ti atoms as opposed to a binding a single Ti atom, as prior studies have shown that stable adsorption typically involves interactions with multiple surface sites to minimize the distortion of the adsorbed molecule.^17^ In addition to these binding modes, adsorption can occur either molecularly or dissociatively (Figure 3), with the latter involving cleavage of one or more O–H bonds and proton transfer to neighboring bridging oxygen (O_b_) atoms. Due to the asymmetry of the hydrated surface, four distinct bridging oxygen (O_b_) sites are available to accommodate the dissociated protons; these are labeled a–d in Figure 2. Multiple adsorption configurations of boric acid were modeled on the hydrated surfaces, sampling various orientations for each binding mode, including monodentate, bidentate, molecular, and dissociated structures. A subset of the most stable boric acid configurations on each surface model was then used as a basis for modeling the adsorption of the other boronic acids.

**Figure 3:**
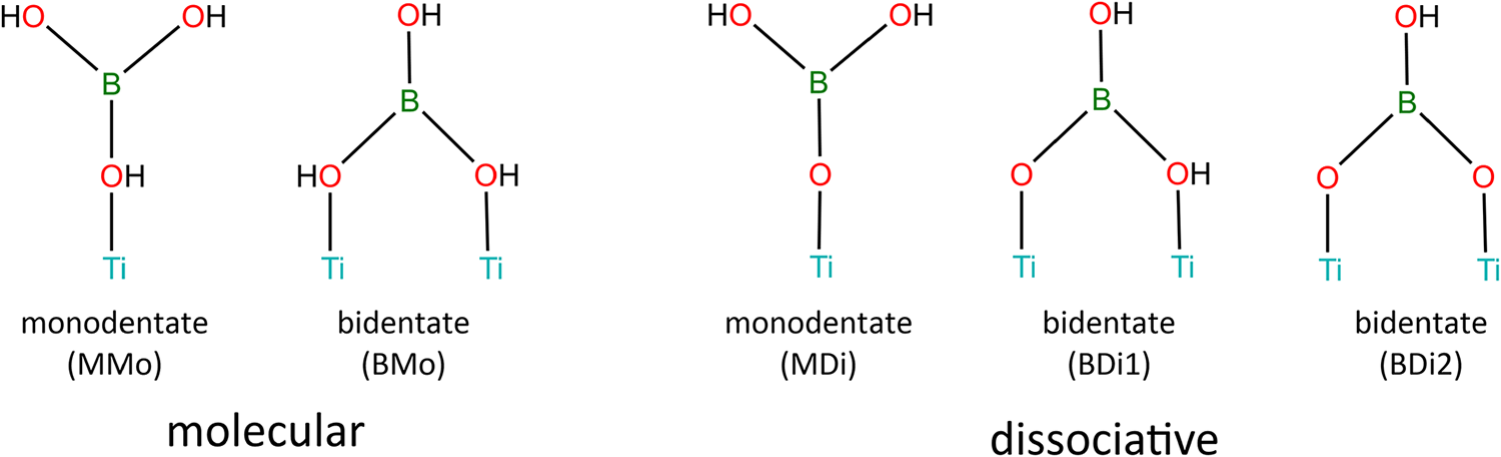
Monodentate and bidentate adsorption configurations of boric acid on TiO_2_, for both molecular and dissociative adsorption.

The set of boronic acids investigated in this study is identical to that used in our previous study of adsorption on the clean TiO_2_ surface,^10^ and is shown in Figure 4. To model the isolated molecules, each boronic acid was placed in a vacuum cell measuring 20 Å × 20 Å × 20 Å. Boronic acids can adopt either cis or trans conformations, with the trans form illustrated for boric acid in Figure 4a. Only the trans conformation was considered in this study, as it is energetically favored over the cis form by approximately 4.8 kcal/mol.^11,18,19^ For the adsorption of 2-FPBA, we placed the fluorine atom on either side of the phenyl ring. Sampling the directionality of substitution is important for 2-FPBA because the close position of the fluorine to the boronic acid binding group can more directly influence the molecule’s steric interactions and electronic environment at the binding site. In contrast, for 3-FPBA and 4-FPBA, the fluorine is more spatially removed from the anchoring group, making its orientation less likely to affect adsorption geometry or energetics.

**Figure 4:**
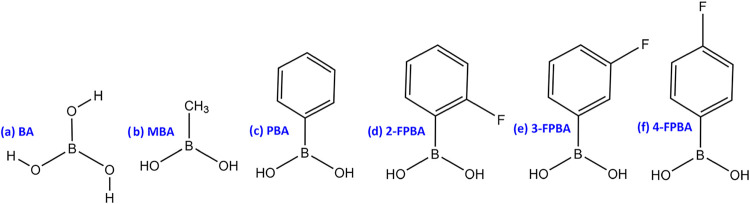
Functionalized boronic acids considered in this study: (a) boric acid (BA), (b) methylboronic acid (MBA), (c) phenylboronic acid (PBA), (d) 2-fluorophenylboronic acid (2-FPBA), (e) 3-fluorophenylboronic acid (3-FPBA), (f) 4-fluorophenylboronic acid (4-FPBA).

### Calculation Details and Analyses

All calculations were performed using the Vienna Ab Initio Simulation Package (VASP).^20–25^ The Perdew–Burke–Ernzerhof (PBE) exchange-correlation functional^26,27^ was employed, with DFT + *U* corrections applied to Ti 3d states using Dudarev’s formalism,^12^ and with a *U*–*J* value of 4.2 eV to ensure proper charge localization, consistent with prior studies.^28–33^ Dispersion interactions were accounted for using Grimme’s DFT-D3 correction.^13,14^ Projector augmented-wave (PAW) potentials provided with VASP were used to replace the core electrons. Brillouin zone sampling was performed using a Γ-centered 2 × 2 × 1 mesh with Gaussian smearing of 0.05 eV. A plane-wave kinetic energy cutoff of 700 eV was applied, which converged the total energy to within 1 meV per atom. Structural optimizations were conducted with a force convergence criterion of 0.01 eV·Å^–1^. All calculations included spin polarization.

The adsorption energy of a boronic acid (BA) molecule was calculated using the equation

1 $$E_{\mathrm{ads}}=E_{\mathrm{BA}/{\mathrm{TiO}}_{2}\cdot \mathrm{hyd}}-E_{\mathrm{BA}}-E_{{\mathrm{TiO}}_{2}\cdot \mathrm{hyd}}+\Delta \mathrm{ZPE}$$

where *E*_BA/TiO_2_·hyd_ is the total energy of the boronic acid adsorbed on the hydrated TiO_2_ surface, *E*_BA_ is the energy of the isolated boronic acid molecule calculated in the vacuum cell, and *E*_TiO_2_·hyd_ is the energy of the hydrated TiO_2_ surface. The ΔZPE accounts for the zero-point energy (ZPE) corrections and is defined as

2 $$\Delta \mathrm{ZPE}={\mathrm{ZPE}}_{\mathrm{BA}/{\mathrm{TiO}}_{2}\cdot \mathrm{hyd}}-\mathrm{ZP}E_{\mathrm{BA}}-{\mathrm{ZPE}}_{{\mathrm{TiO}}_{2}\cdot \mathrm{hyd}}$$

Although ZPE contributions are often omitted in adsorption energy calculations (including previous studies of boric acid on TiO_2_ ^11,19^ ), they provide essential corrections for improved accuracy, even if small compared to electronic energies. The ZPE of any slab or molecule is calculated as

3 $$\mathrm{ZPE}=\frac{1}{2}\sum_{i}h\nu_{i}$$

The vibrational frequencies (ν_*i*_) were computed by evaluating the numerical Hessian using a finite difference approach, with atomic displacements of 0.01 Å in each Cartesian direction. Because vibrational changes upon BA adsorption are expected to be localized near the binding site, only surface atoms within 4 Å of the adsorbed molecule were included in ZPE calculations for the surface. This approximation allows direct evaluation of the difference ZPE_BA/TiO_2_·hyd_– ZPE_TiO_2_·hyd_, rather than computing full vibrational spectra for all atoms in the slab. Additionally, vibrational frequency analysis confirmed the structural stability of optimized configurations by verifying the absence of imaginary frequencies.

The adsorption energy of water on clean TiO_2_ was calculated similarly using the equation

4 $$E_{\mathrm{ads}}=E_{{\mathrm{TiO}}_{2}\cdot \mathrm{hyd}}-E_{H_{2}O}-E_{{\mathrm{TiO}}_{2}}+\Delta \mathrm{ZPE}$$

where *E*_TiO_2_·hyd_ is the total energy of the hydrated TiO_2_ surface, *E*_H_2_O_ is the total energy of the isolated water molecule, and *E*_TiO_2__ is the total energy of the clean TiO_2_ surface.

Bader charge analysis^34^ was performed to investigate changes in the electronic structure of surface atoms upon hydration. Bader analysis partitions the charge density by identifying zero-flux surfaces that define distinct “Bader volumes” for each atom; the total charge integrated within each volume approximates the electronic charge associated with that atom. Bader charges were calculated for representative models of both the clean and hydrated surfaces, with a focus on the surface Ti and O atoms at the adsorption active site. This approach enabled the evaluation of how dissociative water adsorption affects charge density on the surface and influences subsequent boronic acid binding.

The surface energy of the hydrated surface was calculated as

5 $$\gamma^{\prime }=\gamma +\frac{nE_{\mathrm{ads}}}{A^{\prime }}$$

where γ is the surface energy of the clean surface, *n* is the number of adsorbed water molecules (1 in our case), *E*_ads_ is the adsorption energy of the dissociated water (which is expected to be a negative value for exothermic adsorption, based on eq 4), and A’ is the surface area of one surface of the slab.

## Results and Discussion

### Adsorption Energy of Water

The five hydrated TiO_2_(110) surfaces were first optimized without the boronic acid adsorbate to establish a stable reference for boronic acid adsorption. To compare the relative stabilities of these configurations, we calculated the adsorption energies of dissociated water for each structure (Table 1). These adsorption energies correspond to an average surface energy of 1.28 J/m^2^ for the hydrated surface, in contrast with the surface energy of clean rutile (110) of 1.43 J/m^2^.^10^ The resulting adsorption energies vary modestly, within a range of approximately 3.8 kcal/mol. The differences between configurations arise from the relative positions of the adsorbed OH^–^ and H^+^ species, and from the orientations of hydrogen atoms on the surface. Due to the inherent symmetry of the clean TiO_2_ surface without the boronic acid adsorbate, some configurations share equivalent positions for their adsorbed water fragments. Specifically, configurations A1, B1, and B2 exhibit similar placements of the dissociated species, as do A2 and A3 (see Figure 2). Within each group, however, the orientation of the surface hydrogen atoms most significantly affects the adsorption energy. The enhanced stability of the A3 configuration is due to a hydrogen-bonding interaction in which the adsorbed H^+^ is oriented toward the oxygen atom of the OH^–^ group. In contrast, less stable configurations (A1 and A2) show the hydrogen of the OH group oriented toward the H^+^, likely leading to repulsive interactions that reduce the binding strength.

**Table 1:** Adsorption Energy (kcal/mol) of Dissociated Water on the Rutile TiO_2_(110) Surface, with ZPE Corrections

configuration	*E*_ads_ (kcal/mol)
A1	–32.93
A2	–31.48
A3	–35.25
B1	–34.06
B2	–34.76

There had been some difficulty in optimizing these configurations, so the initial structures were constructed by placing the H atoms vertically atop the surface O atoms, allowing the optimization process to determine the final orientation. Therefore, the diversity in hydrogen bonding observed across the configurations emerged organically from the relaxation process, rather than being artificially imposed. This natural variation offers a realistic sampling of plausible surface hydration environments, which is valuable for averaging over differences in local hydroxyl group arrangements that influence subsequent boronic acid adsorption.

### Adsorption of Boric Acid

The same adsorption geometries previously examined on the clean rutile TiO_2_(110) surface were considered here for the hydrated surface.^10^ A selection of their optimized configurations on the A1 hydrated model is presented in Figure 5. In the monodentate molecular configuration (MMo, Figure 5a), the adsorbed boric acid aligns along the [11̅0] direction, enabling its acidic hydrogen atoms to form hydrogen bonds with adjacent bridging oxygen (O_b_) atoms, thereby stabilizing adsorption. Only the monodentate coordination is feasible in this direction, due to the absence of two adjacent Ti_5c_ atoms along this direction. The monodentate configuration can also dissociate to form the monodentate dissociative configuration (MDiA, Figure 5b), in which the H bound to the adsorbed O cleaves and binds to its nearest neighbor O_b_.

**Figure 5:**
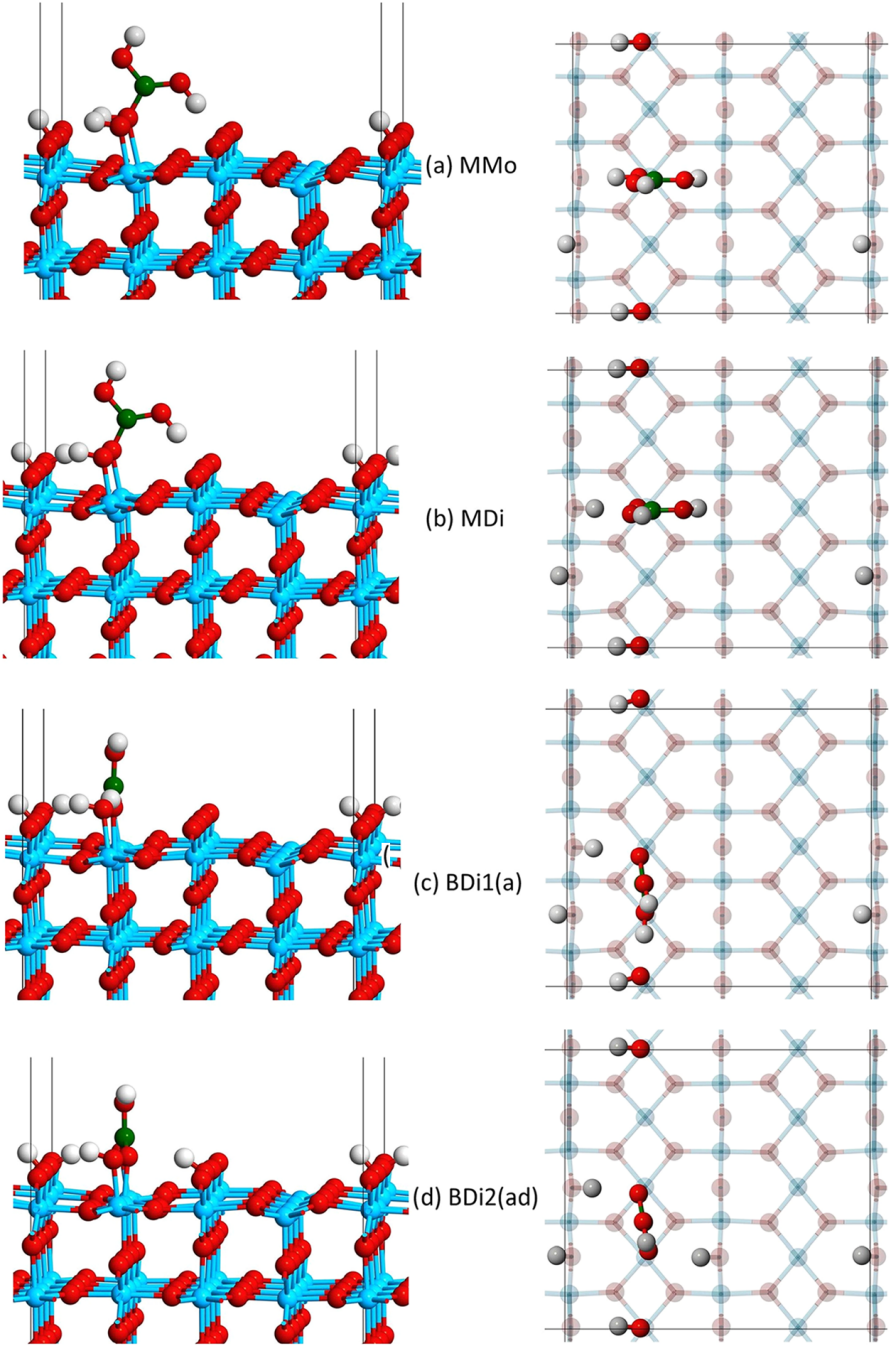
Representative configurations of boric acid adsorbed on the hydrated A1 TiO_2_ surface: (A) monodentate molecular (MMo), (B) monodentate singly dissociated (MDi), (C) bidentate singly dissociated with protonation at site a (BDi1­(a)), and (D) bidentate doubly dissociated with protonation at sites a and d (BDi2­(ad)).

The bidentate configuration is oriented in the [001] direction, so that the molecule can access two neighboring Ti_5c_ atoms. There was no bidentate molecular structure found to be stable on the hydrated surface, so this configuration is not considered. When one hydrogen dissociates (BDi1, Figure 5c), the hydrogen dissociates from either of the adsorbed oxygen atoms to bind to one of the four neighboring O_b_ atoms, labeled a–d in Figure 2. This results in four unique BDi1 structures, denoted in their naming as a–d (e.g., BDi1­(a)). In the doubly dissociated bidentate configuration (BDi2, Figure 5d), the two dissociated hydrogens can bind on either side of the adsorbate, resulting in four possible configurations (ab, bc, ad, and cd). Figure 5 shows one representative example for each of the BDi1 and BDi2 structures, but there are a total of four possible configurations for each. In the A1 surface, the *b* position is already occupied by the H^+^ from the dissociated water, so the configuration ab and bc were not considered there. All of these configurations were used to examine the adsorption of boric acid on the hydrated rutile TiO_2_ (110) surface.

The contribution of zero-point energy (ZPE) corrections to the total adsorption energy is small, at less than 2% of the electronic energy component. As a result, including the ZPE does not meaningfully impact the overall energetic trends. Due to the high computational cost of ZPE calculations, adsorption energies for boric acid were initially computed without the ΔZPE term to facilitate the preliminary identification of the most favorable adsorption configurations.

Table 2 presents the adsorption energies for each configuration, averaged over the five hydrated surface models and four dissociative binding sites (a–d). For comparison, previously reported adsorption energies on the clean rutile TiO_2_ (110) surface are also included.^10^ (Individual adsorption energies for each configuration on each hydrated surface, still without ZPE corrections, are provided in Supporting Information Table S1. The corresponding surface-averaged values are shown in Table S2).

**Table 2:** Adsorption Energies (kcal/mol) of Boric Acid Averaged on all Hydrated TiO_2_ Surfaces, without ZPE Corrections, in Comparison with the Clean Surface^10^

adsorbate	clean TiO_2_(110)	hydrated TiO_2_(110)
MMo	–36.46	–29.52
MDi	–37.61	–32.36
BDi1(avg)	–51.81	–44.45
BDi2(avg)	–55.46	–48.82

On the hydrated surfaces, the doubly dissociated configuration (BDi2) remains the most stable adsorption mode, consistent with trends observed on the clean surface. However, the presence of surface water generally leads to less favorable adsorption, as indicated by the reduced adsorption energies (i.e., less negative) compared to those on the clean surface. As shown in Table S2, there is a notable variation in adsorption energies across the different hydrated surfaces, emphasizing the influence of the proximity of the preadsorbed water to the adsorbed boric acid molecule. This variability highlights the importance of sampling multiple hydration structures to obtain a representative average and better reflect the range of realistic adsorption environments.

### Adsorption of Boronic Acids

A total of 44 adsorption models were evaluated for boric acid, representing all combinations of its adsorption configurations across the five hydrated surface models. To reduce the computational cost for the remaining boronic acids, we selected only the three most stable configurations for each hydrated surface based on the boric acid results. For example, on the hydrated A2 surface, only the BDi1­(d), BDi2­(ad), and BDi2­(cd) geometries were considered for the other boronic acids. By focusing on the most favorable and realistic adsorption modes, this approach reduced the number of required models to 15 per boronic acid. To improve the accuracy of these adsorption energies, zero-point energy (ZPE) corrections were included in this final evaluation.

Table 3 presents the adsorption energies, including zero-point energy (ZPE) corrections, for each boronic acid, averaged across all 15 structural models. For 2-FPBA, results are shown in two rows to reflect the placement of the fluorine atom on either side of the phenyl ring, labeled R (right) and L (left). (Individual adsorption energies for each configuration on the hydrated surfaces are listed in Tables S3–S7 in Supporting Information.) Functionalization of the boronic acid enhances adsorption stability in these optimal configurations, with 3-FPBA and 4-FPBA showing the strongest adhesion. These trends are consistent with those observed on the clean surface, suggesting that the relative influence of substituents on binding strength is largely preserved in the presence of surface hydration. However, the increase in magnitude of the adsorption energy with functionalization is less on the hydrated surface, indicating that hydration partially reduces the sensitivity of adsorption strength to chemical substitution.

**Table 3:** Adsorption Energies (kcal/mol) of Each Boronic Acid Averaged over all Five Hydrated Surfaces, with ZPE Corrections, Compared to Values on the Clean Surface^10^

adsorbate	clean	hydrated
BA	–54.59	–49.05
MBA	–55.53	–49.97
PBA	–57.24	–50.10
2-FPBA(R)	–54.75	–48.10
2-FPBA(L)	–54.75	–49.66
3-FPBA	–58.09	–51.17
4-FPBA	–58.83	–51.08

### Bader Charge Analysis

To better understand the origin of the differences in adsorption strength observed between the clean and hydrated TiO_2_ surfaces, we performed a Bader charge analysis of the clean TiO_2_ surface and all hydrated models. Changes in atomic charge reflect changes in local electronic structure caused by surface hydration and provide insight into how these changes affect molecular adsorption.

Table 4 shows the Bader charges of the surface atoms at the adsorption site for the clean TiO_2_ surface and hydrated TiO_2_ models (O_b_ atoms are labeled a-d as in Figure 2, and the Ti_5c_ atoms are now numbered). There are a few key differences between the charges of the clean and hydrated surfaces. First, the surface oxygen atoms that bind a dissociated proton to form an adsorbed hydroxyl on the surface (i.e., A1-O­(b), B1–O­(a)) become significantly more negative by about 0.261 e^–^. In the B models, the O­(d) atom also has increased electron density (by 0.055 e^–^), due to the neighboring hydroxyl group. The increase in electron density is indicative of passivation, that these oxygens are either already bound to H directly or partially passivated via hydrogen-bonding to the nearby hydroxyl. This polarizes the oxygens and makes them less available to stabilize new hydrogen bonds or bind to dissociated hydrogen. The increased negative charge also increases the electrostatic repulsion with the boronic acid oxygens, further reducing adsorption stability.

**Table 4:** Bader Charges (e^–^) of the Active Site Atoms in the Clean Rutile TiO_2_ (110) Surface and the A1 Model of the Hydrated Rutile TiO_2_ (110) Surface

	clean	A1	A2	A3	B1	B2
O(a)	–0.907	–0.905	–0.911	–0.902	–1.168	–0.900
O(b)	–0.907	–1.166	–0.904	–0.901	–0.901	–0.901
O(c)	–0.906	–0.909	–0.902	–0.909	–0.916	–0.916
O(d)	–0.906	–0.907	–0.908	–0.916	–0.961	–0.961
Ti(1)	2.034	2.019	2.018	2.018	2.037	2.037
Ti(2)	2.034	2.019	2.027	2.022	2.036	2.037

On the A surfaces, the local increase in electron density reduces the positive Bader charges of nearby titanium atoms. This reduction in charge decreases the Lewis acidity of surface Ti sites, decreasing their ability to bind with the oxygen atoms in the boronic acids. On the B surfaces, there is no change to the charges on the Ti atoms at the active site, so this effect does not contribute to the reduction of adsorption stability.

Overall, hydrating the surface results in the surface being partially passivated, decreasing the number of undercoordinated surface atoms that would be reactive, and changing the electronic structure of other nearby undercoordinated surface atoms, thereby impacting their ability to bind.

## Conclusions

This study expanded on prior investigations of boronic acid adsorption by incorporating hydration effects on the TiO_2_ rutile (110) surface. Using an ensemble of low-coverage hydrated surface models, we demonstrated that boric acid adsorbs in the bidentate doubly dissociative configuration, consistent with optimal adsorption on the clean surface, although adsorption is weakened by hydration. Functionalized boronic acids, particularly fluorophenyl derivatives, still exhibit enhanced adsorption stability, though the difference in binding strength between functional groups is slightly reduced on the hydrated surface.

Electronic structure analysis through Bader charge partitioning revealed that surface hydration modifies the charges at the active site, lowering the positive charge on surface Ti atoms. This charge redistribution slightly weakens the interaction between the surface and adsorbates, also lessening the impact of molecular functionalization. The adsorbed water also creates a dipole moment that reduces the surface potential, thus lowering the work function, weakening the surface’s ability to bind other molecules.

The consistency of binding preferences across clean and hydrated surfaces validates prior studies as a benchmark for intrinsic binding trends, while the hydrated surface models better reflect realistic interfacial conditions. Together, these findings support the potential of boronic acid derivatives as viable anchoring groups in DSSCs. While this study has focused on ideal defect-free TiO_2_ surfaces, it is well established that oxygen vacancies are a common feature under experimental conditions and can strongly influence adsorption. By first establishing adsorption trends on pristine and hydrated surfaces, this work provides a critical baseline against which the effects of point defects such as oxygen vacancies can be systematically investigated in future studies.

## Supplementary Material



## Notes

Tables providing the individual adsorption energies for each configuration on each hydrated surface (PDF)
