# Supplementary material for: Boronic Acid Adsorption on Hydrated Rutile TiO2(110): A DFT + U Study
Source: ACS Omega. 2025 Nov 13;10(46):55942–9. doi: 10.1021/acsomega.5c07528 (PMC12658694; doi:10.1021/acsomega.5c07528)
Supplement: Supplementary file 1 [file ao5c07528_si_001.pdf]

Supporting Information for

Boronic acid adsorption on hydrated rutile TiO_2 (110): A DFT+U study

Leah Isseroff Bendavid,* Julie Geller

Department of Chemistry, 124 Raymond Ave, Box 175, Vassar College, Poughkeepsie, New York, 12604

*email: lebendavid@vassar.edu, phone: 845-437-5993

Table S1: Adsorption energies (kcal/mol) of boric acid on each hydrated TiO_2 surface without ZPE corrections

Adsorbate	A1	A2	A3	B1	B2
MMo	-30.19	-30.29	-30.59	-25.58	-30.92
MDi1	-29.92	-29.62	-33.33	-33.07	-35.88
BDi1(a)	-41.14	-44.07	-48.97	-47.25	-44.69
BDi1(b)	N/A[1]	-35.20	-46.91	-44.92	-45.62
BDi1(c)	-45.45	-48.26	-45.72	N/A	-39.15
BDi1(d)	-43.44	-49.63	-43.93	-43.48	-43.43
BDi2(ab)	N/A	-38.63	-49.03	-51.49	-50.32
BDi2(ad)	-45.76	-55.22	-50.37	-53.49	-51.51
BDi2(bc)	N/A	-45.61	-50.59	N/A	-50.77
BDi2(cd)	-46.20	-54.05	-44.77	N/A	-45.62

[1]Entries of N/A correspond to configurations that do not exist to avoid placing two hydrogens on the same oxygen.

Table S2: Adsorption energies (kcal/mol) of boric acid in each configuration, averaged on all hydrated TiO_2 surfaces, without ZPE corrections

Adsorbate	Hydrated	Clean
MMo	-29.52	-33.46
MDi1	-32.36	-37.61
BDi1(a)	-45.22	-51.81
BDi1(b)	-43.16	-51.81
BDi1(c)	-44.65	-51.81
BDi1(d)	-44.78	-51.81
BDi2(ab)	-47.36	-55.46
BDi2(ad)	-51.27	-55.46
BDi2(bc)	-48.99	-55.46
BDi2(cd)	-47.66	-55.46

Table S3: Adsorption energies with ZPE corrections (kcal/mol) of the boronic acids in their three most stable configurations on the hydrated A1 surface

Adsorbate	BDi2(cd)	BDi2(bc)	BDi1(c)
BA	-46.55	-45.80	-44.50
MBA	-46.90	-45.78	-45.09
PBA	-49.54	-46.87	-43.03
2-FPBA (R)	-45.82	-43.08	-44.96
2-FPBA (L)	-48.78	-46.63	-43.09
3-FPBA	-49.92	-47.63	-44.02
4-FPBA	-50.08	-47.76	-44.33

Table S4: Adsorption energies with ZPE corrections (kcal/mol) of the boronic acids in their three most stable configurations on the hydrated A2 surface

Adsorbate	BDi2(cd)	BDi2(bc)	BDi1(d)
BA	-53.44	-45.16	-49.52
MBA	-53.37	-46.12	-50.89
PBA	-53.20	-46.71	-49.22
2-FPBA (R)	-50.65	-43.08	-44.96
2-FPBA (L)	-53.18	-54.39	-48.64
3-FPBA	-54.20	-48.30	-49.49
4-FPBA	-54.27	-48.05	-50.25

Table S5: Adsorption energies with ZPE corrections (kcal/mol) of the boronic acids in their three most stable configurations on the hydrated A3 surface

Adsorbate	BDi2(bc)	BDi2(ad)	BDi2(ab)
BA	-49.72	-49.65	-47.45
MBA	-51.26	-51.32	-49.49
PBA	-51.28	-51.36	-49.57
2-FPBA (R)	-49.06	-48.60	-47.65
2-FPBA (L)	-50.12	-50.65	-49.20
3-FPBA	-53.05	-52.62	-50.52
4-FPBA	-52.46	-53.25	-50.74

Table S6: Adsorption energies with ZPE corrections (kcal/mol) of the boronic acids in their three most stable configurations on the hydrated B1 surface

Adsorbate	BDi2(ad)	BDi2(ab)	BDi1(a)
BA	-53.01	-51.37	-46.58
MBA	54.65	-52.72	-46.56
PBA	-54.37	-52.80	-46.02
2-FPBA (R)	-52.99	-50.67	-43.24
2-FPBA (L)	-53.87	-49.38	-48.33
3-FPBA	-56.92	-54.21	-47.38
4-FPBA	-56.07	-53.07	-50.74

Table S7: Adsorption energies with ZPE corrections (kcal/mol) of the boronic acids in their three most stable configurations on the hydrated B2 surface

Adsorbate	BDi2(ad)	BDi2(bc)	BDi2(ab)
BA	-51.72	-51.04	-50.26
MBA	-52.50	-51.66	-51.20
PBA	-52.88	-53.22	-51.41
2-FPBA (R)	-49.54	-49.90	-49.33
2-FPBA (L)	-49.32	-49.65	-49.64
3-FPBA	-53.27	-53.08	-52.90
4-FPBA	-53.92	-53.57	-52.57
